# Current progress in chimeric antigen receptor T cell therapy for glioblastoma multiforme

**DOI:** 10.1002/cam4.4064

**Published:** 2021-06-19

**Authors:** Hany E. Marei, Asmaa Althani, Nahla Afifi, Anwarul Hasan, Thomas Caceci, Giacomo Pozzoli, Carlo Cenciarelli

**Affiliations:** ^1^ Department of Cytology and Histology Faculty of Veterinary Medicine Mansoura University Mansoura Egypt; ^2^ Biomedical Research Center Qatar University Doha Qatar; ^3^ Qatar Biobank Doha Qatar; ^4^ Department of Mechanical and Industrial Engineering College of Engineering Qatar University Doha Qatar; ^5^ Biomedical Sciences Virginia Maryland College of Veterinary Medicine Blacksburg Virginia USA; ^6^ Pharmacology Unit Fondazione Policlinico A. Gemelli IRCCS Rome Italy; ^7^ Institute of Translational Pharmacology‐CNR Rome Italy

**Keywords:** chimeric antigen receptor (CAR) T cell, clinical trials, FcγRs CAR‐T cells, GBM‐associated antigens, glioblastoma multiforme (GBM)

## Abstract

Glioblastoma multiforme (GBM) is one of the deadliest brain tumors with an unfavorable prognosis and overall survival of approximately 20 months following diagnosis. The current treatment for GBM includes surgical resections and chemo‐ and radiotherapeutic modalities, which are not effective. CAR‐T immunotherapy has been proven effective for CD19‐positive blood malignancies, and the application of CAR‐T cell therapy for solid tumors including GBM offers great hope for this aggressive tumor which has a limited response to current treatments. CAR‐T technology depends on the use of patient‐specific T cells genetically engineered to express specific tumor‐associated antigens (TAAs). Interaction of CAR‐T cells with tumor cells triggers the destruction/elimination of these cells by the induction of cytotoxicity and the release of different cytokines. Despite the great promise of CAR‐T cell‐based therapy several challenges exist. These include the heterogeneity of GBM cancer cells, aberrant various signaling pathways involved in tumor progression, antigen escape, the hostile inhibitory GBM microenvironment, T cell dysfunction, blood‐brain barrier, and defective antigen presentation. All need to be addressed before full application at the clinical level can begin. Herein we provide a focused review of the rationale for the use of different types of CAR‐T cells (including FcγRs), the different GBM‐associated antigens, the challenges still facing CAR‐T‐based therapy, and means to overcome such challenges. Finally, we enumerate currently completed and ongoing clinical trials, highlighting the different ways such trials are designed to overcome specific problems. Exploitation of the full potential of CAR‐T cell therapy for GBM depends on their solution.

## Graphical abstract

Here, we provided a focused review on the rationale of the use of different types of CAR‐T cells including FcγRs CAR‐T cells, different GBM‐associated antigens, challenges still facing CAR‐T‐based therapy for GBM, and strategies to overcome such challenges. Finally, we enumerated the completed and ongoing clinical trials for GBM and highlighted the different ways such trials are designed to overcome specific challenges that guard against the exploitation of the full potential of CAR‐T cell therapy for GBM.

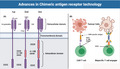

## 1 INTRODUCTION

The chemo‐, radio‐resistant, and recurrent nature of glioblastoma multiforme (GBM) make it one of the deadliest forms of high‐grade gliomas. It has an average incidence of 4.67 to 5.73 per 100,000 people,^1^ and overall survival of 20 months.^2^, ^3^ The inability of most drugs to cross the blood–brain barrier (BBB) further complicates treatment and reduces the efficacy of different available standard of care (SOC) modalities. Future therapies for GBM need to overcome specific challenges: barriers to immune cells, defective antigen presentation, and T cell function impairments.^3^

Adoptive cell therapy (ACT) with chimeric antigen receptor (CAR) T cells is expected to overcome challenges associated with GBM treatment. It may not only provide more effective targeted therapeutic strategy against specific tumor‐associated antigens (TAA), but also increase the ability of activated T cells to overcome the BBB.^4^, ^5^, ^6^

CAR‐T cell therapy for GBM is developed to specifically target certain GBM‐associated antigens which are not or minimally expressed in normal brain tissues. Currently, the safety and efficacy of several specific GBM antigens are being tested at preclinical and clinical levels. These include interleukin‐13 receptor alpha 2 (IL13Rα2),^7^ human epidermal growth factor 2 (HER2),^8^ erythropoietin‐producing hepatocellular carcinoma A2 (EphA2),^9^ ganglioside 2 (GD2),^10^ B7‐H3,^11^ and chlorotoxin.^12^ Despite promising outcomes at the preclinical level, the clinical efficacy of these immunotherapeutic modalities is not optimal. Increasing the efficacy of ACT for GBM would require combination therapy that includes not only chemo‐ and radio‐therapeutic approaches but also needs to be integrated with other recent immunotherapeutic approaches.^13^ Effective combination therapy would be needed to produce a more effective, safer, and more specific therapeutic regime to be added to the existing SOC. In this review, we will provide a synopsis of the rationale for the use of different types of CAR‐T cells (including FcγRs CAR‐T cells), different GBM‐associated antigens, the challenges still facing CAR‐T‐based therapy, and how to overcome such challenges. We will also provide a synopsis of T cell trafficking within brain tissues, with emphasis on the mechanism by which immune cells might cross the BBB. Finally, we discuss the potential of ongoing clinical trials and how such therapeutic approaches may revolutionize the current SOC for GBM.

## 2 CAR‐T CELLS TECHNOLOGY

The way in which T lymphocytes are activated against foreign antigens involves the interaction of several integral molecules/receptors existing on their surfaces. Specifically, T cell receptors (TCR) are the key inducers of T cell activation. Interaction of TCRs with foreign antigens presented in association with major histocompatibility class I molecules induces T cells to become activated and able to destroy the antigens that provoked their activation. The highly specific nature of TCR action against antigens and the unique structure of TCR has attracted researchers to mimic TCR function as a way to generate specific T cells directed against cancer cells.^14^

CAR‐T technology was developed in the last three decades. It is based on the generation of genetically engineered T lymphocytes directed to recognize and eliminate specific TAA. The basic structure of the CAR constructs involves three main domains: the extracellular, the transmembrane/linker/spacer, and the intracellular ones. Currently, there are at least different generations of CAR‐T cells, which are continuously being improved in terms of specificity and persistence within tumor microenvironments.

The basic structure of CAR‐T constructs consists of a targeting moiety (which in most cases involves a single‐chain fragment variable (scFv) from a monoclonal antibody) connected to a spacer domain, a transmembrane region, and an intracellular CD3ζ chain (the signaling domain of a TCR).^15^ The unique construction of CAR‐T molecules not only allows the identification of a wide range of antigens but also works independently of major histocompatibility complex presentation, which often is downregulated by tumor cells.^16^

Binding to specific antigens triggers the CAR‐T cells activation. This then brings about the elimination of the provoking antigens. Elimination occurs through the release of cytokines, perforin, and cytotoxic degranulation.^17^ First‐generation CAR‐T cells (which utilized CD3ζ chain as an intracellular activation domain) were efficient in the elimination of the tumor burden at the preclinical levels but had only limited efficacy at the clinical level. They were incompletely ineffective in human patients,^18^ mainly due to their poor persistence within the hostile tumor microenvironment.^19^

**FIGURE 1:**
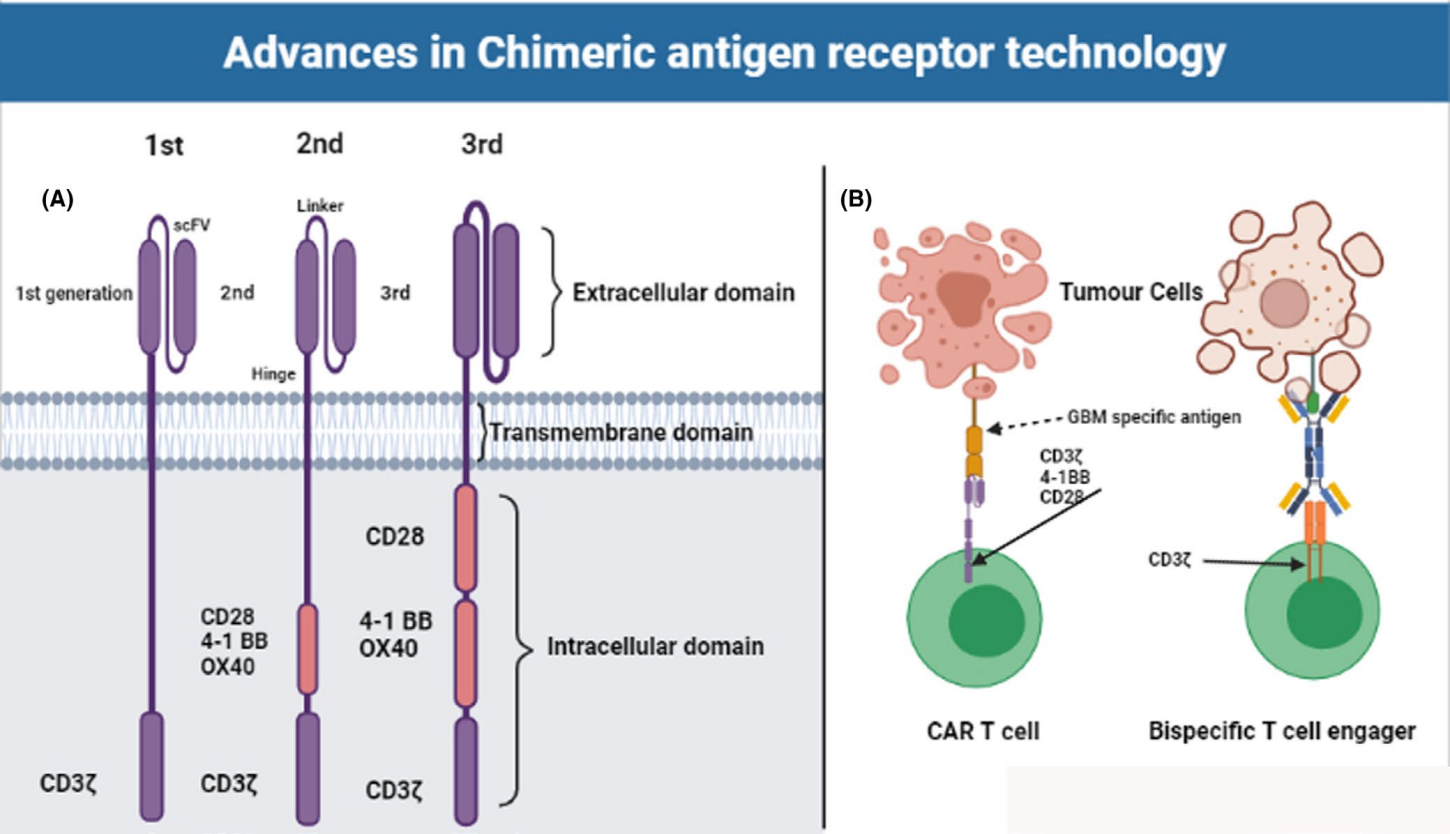
Basic CAR‐T cells technology and recent improvement including the differences between CAR‐T and bispecific T cell engager

In attempts to increase tumor‐elimination power the second, third, and fourth generations, CAR‐T cells were designed which include costimulatory domains such as CD28, OX40, and 41BB added to the basic CD3ζ (Figure 1). To further increase potency against malignant cells, CAR‐T cells were generated to include additional proteins (such as cytokine homing receptors, or other biologics).^20^

FDA approval for the utilization of CD19 CAR‐T cells for relapsed or refractory B‐cell lymphomas including cases that involve CNS disease ^4^ stimulated the generation of novel CAR designs and targets intended to achieve superior performance.^21^ The route from bench to bedside application was hindered by many challenges: commercial accessibility, safety, and resistance. All of these are critical issues for any CAR‐T cell therapy. While CAR‐T cell therapy is approved for hematological malignancies, its potential has not yet been fully realized for the treatment of GBM.

## 3 MAJOR CHALLENGES FOR GBM CAR‐T CELLS THERAPY

Several major challenges for application to GBM still exist. Whether or not trafficking of immune cells such as CAR‐T cells is allowed through the BBB is still in need of further examination. Previous studies clarified that immune cells have only limited access to the brain due to the presence of the BBB and the lack of resident dendritic cells^22^ but recent studies have elucidated that activated T cells can pass the BBB and diffusely penetrate the brain parenchyma.^4^, ^5^, ^6^ Several strategies for enhancing homing of T cells from the blood to the brain have been suggested. These include VLA‐4 and CXCR10 expressions, which influence T cell homing to the CNS.^23^ Intraventricular delivery of CAR‐T cells enables the trafficking of CAR‐T cells to tumor sites, as well as other distant tumor cells.^24^ CAR‐T cells have been detected in GBM samples after intratumoral and intravenous infusion.^23^ Further investigations to determine the ideal site of administration of CAR‐T cell for GBM are still needed.^25^

The second major challenge is the persistence of CAR‐T cells within the GBM environment. Previous studies have demonstrated that CAR‐T cell persistence was enhanced following lymphodepletion with chemotherapy in blood malignancies.^26^ The effect of lymphodepletion on EGFRvIII targeting CAR‐T cells for GBM is being assessed in an ongoing clinical trial in which patients are subjected to fludarabine and cyclophosphamide chemotherapy before T cell infusion and IL2 after infusion (NCT 01454596). Combined treatment with CAR‐T cells and chemoradiotherapy may provide a much more effective therapeutic regime for early GBM.^27^

The hostile and inhibitory microenvironment of GBM is another major challenge to CAR‐T therapy. The activity of CAR‐T cells may be suppressed by the presence of inhibitory cell‐surface antigens such as programmed death 1/programmed death‐ligand 1 (PD‐1/PD‐L1)^22^, ^24^; or by releasing immunosuppressive cytokines such as prostaglandin E2, IL‐10, and TGFβ.^23^ Trafficking of immunosuppressive cells^27^ is also a decisive factor that creates other physical and metabolic blockades.^28^ The use of anti‐PD‐1 and anti‐PD‐L1 antibodies at the preclinical level provided encouragement to combine immune checkpoint inhibitors (ICPIs) with EGFRvIII CAR‐T cells.^29^ Increased antitumor responses have been reported in blood lymphoma after CD19 CAR‐T cell therapy and pembrolizumab infusion.^30^

Finally, providing a superb CAR‐T therapy for GBM might be challenged by antigen downregulation/escape and different tumor cell composition. Targeting multiple GBM‐associated antigens using bi‐ and tri‐specific CAR‐T cells has been found to increase the efficacy of CAR‐T cells.^28^, ^31^

Currently, the standard treatment for GBM includes corticosteroids and chemotherapy. CAR‐T‐based therapy for GBM is directed against several GBM‐associated antigens such as IL13Rα2,^32^, ^33^ EGFRvIII,^34^ and HER2.^35^ Early clinical trials have shown a positive result from the use of the CAR‐T cells as an additional therapy for GBM, which encourages future work in this research area.

## 4 EFFICACY OF CAR‐T FOR GBM

### 4.1 IL‐13Rα2 CAR‐T Cells

IL‐13Rα2 is one of the GBM‐associated antigens. It is expressed in over 75% of GBM^34^ patients. These patients usually have a dismal prognosis^36^ and activation of IL‐13Rα2 is associated with an increase in the invasiveness of GBM. This comes about through the activation of several molecular targets for the rapamycin pathway.^37^ The safety and efficacy of IL‐13Rα2 CAR‐T were examined by Brown et al. who directly injected IL‐13 CAR‐T cells into the tumor cavities post‐surgery. Intratumor implantation of IL‐13 CAR‐T cells has proven to be tolerable with very few if any side effects. Reduction of IL‐13Rα2 tumor cell expression was recorded in one patient, with a significant increase in the volume of necrotic tissue volume.^33^ In another study, IL‐13 CAR‐T cells with a 4‐1BB costimulatory domain were implanted in a patient with recurrent, multifocal GBM following multiple surgical resections. Although the treated site remains tumor‐free, non‐treated lesions increased in size, and leptomeningeal tumors were detected by imaging. Intraventricular infusions via lateral ventricles of 10 additional doses of 1 × 10^7^ IL‐13 CAR‐T cells led to the influx of immune cells and an increase in 11 inflammatory cytokines and chemokines. The patients tolerated intracavitary and intraventricular infusions, with complete regression of one tumor. One patient unfortunately had another recurrent GBM, but this study provided crucial data regarding the potential use of CAR‐T cells infused into CSF; and about the activation of host immune responses following local/regionally delivered CAR‐T cell therapy.^32^

### 4.2 HER2 CAR‐T cells

The expression of HER2 in GBM tumors makes it one of the important targeted antigens for CAR‐T cell therapy.^38^ HER2 plays a crucial role in cell proliferation, differentiation, motility, and adhesion, and its overexpression in cancer is usually associated with an unfavorable prognosis.^39^ In a phase I clinical trial, anti‐HER2 CAR‐T cells were systematically administered to 17 HER2‐expressing GBM patients. The HER2‐CAR cells persisted for up to 12 months following infusion. Partial response was detected with a median overall survival (OS) of 11.1 months and progression‐free survival (PFS) of 3.3 months.^35^

### 4.3 EGFRVIII CAR‐T cells

WHO 2016 classification, highlighting a large number of genetic alterations associated with specific GBM phenotypes, has improved the classical histological classification of GBM.^40^ EGFRvIII is constitutively expressed in about 50% of human GBM ^41^ but minimally expressed in normal tissue.^16^ In a phase 1 clinical trial, intravenously delivered second‐generation CAR‐T cells with a humanized anti‐EGFRvIII scFv and 4‐1BB domain were injected into 10 GBM patients. Anti‐EGFRvIII CAR‐T cells were identified in the tumor from four patients who underwent post‐infusion surgery. Significant upregulation of a variety of immunosuppressive molecules (such as indoleamine 2,3‐dioxygenase (IDO) 1, PD‐L1, transforming growth factor (TGF)‐β, and IL‐10) was recorded and significant levels of non‐CAR‐T cells also infiltrated the tissue. These latter included unmodified T cells and immune‐suppressive Tregs. These findings suggest that targeting GBM may induce immunosuppressive effects on the GBM microenvironment.^34^ In another study, EGFRvIII CAR‐T cells were administered intravenously after chemotherapy and supported post‐infusion with low‐dose IL‐2. There was no significant delay in the progression of recurrent GBM.^42^ Optimization for the anti‐EGFRvIII‐targeting CAR‐T cells to treat human GBM is in its early stages and further work is needed.

In a recent study, a tri‐specific CAR‐T cell was generated utilizing the synNotch receptor pathway. EGFRvIII was utilized to replace the extracellular domain of synNotch receptor and was covalently linked to a tandem of IL‐13Rα2‐CD133 CAR. Activation of the tri‐specific CAR‐T cells required dual signals and resulted in a tri‐specific GBM stem cell destruction.^43^, ^44^

### 4.4 Fcγ‐chimeric receptors (Fcγ‐CRS) cell for GBM

**FIGURE 2:**
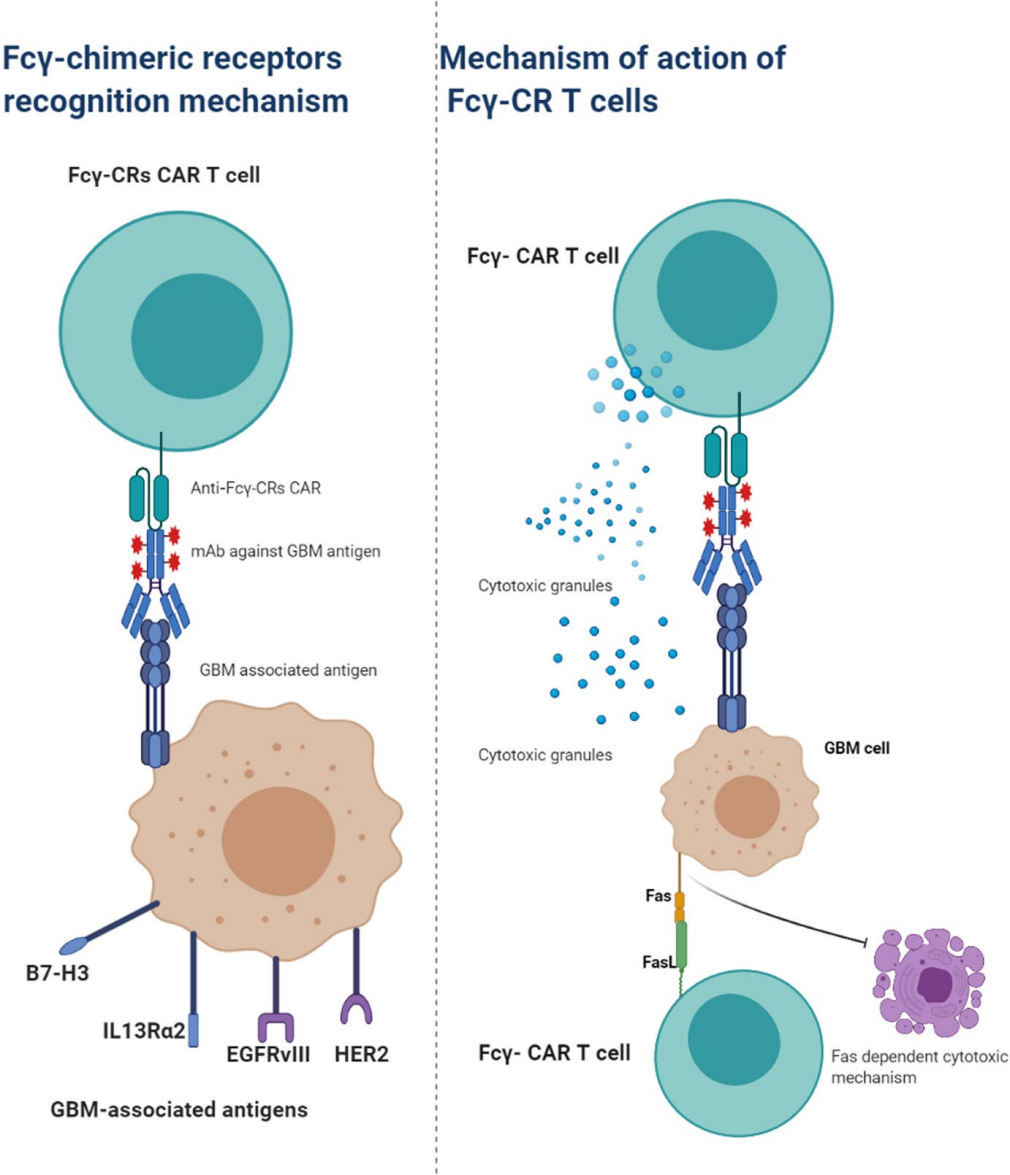
Mechanisms of Fcγ‐CRs T cell‐mediated tumor cell elimination. The Fc portion of monoclonal antibody (mAb) attaches to the Fcγ‐CRs on the surface of Fcγ‐CRs T cells. Following the recognition of GB‐associated antigen and binding of the Fcγ‐CRs T with the mAb, the Fcγ‐CRs T cells are activated leading to the induction of cell‐mediated cytotoxicity either through the release of cytotoxic granules and/or the induction of Fcγ‐CRs dependent FAS expression (lower panel)

In comparison to the conventional CAR construct, the Fcγ‐CRs express the extracellular portion of FcγRs. This will enable the FcγRs CAR‐T cells to attach to the Fc portion of monoclonal antibodies specific against certain TAA. (Figure 2). The transmembrane and stimulatory intracellular domains bear a close similarity in structure to conventional CAR, and likewise, three generations of Fcγ‐CRs T cells are generated (for review about Fcγ‐CRs T cell, see Marei et al.^45^ The rationale behind the use Fcγ‐CRs T cells for cancer immunotherapy is to mimic the function of innate immune cells like NK lymphocytes. The Fcγ‐CRs T cells in combination with mAbs direct the destruction of tumor cells via antibody‐dependent cellular cytotoxicity (ADCC) directed against specific TAA.^46^, ^47^, ^48^ In a previous study by our team, we have assessed, in vitro, the anti‐GBM potential of CD16^158F^‐ and CD16^158V^‐CRs against GBM cancer stem cells (CSC) in the presence or absence of therapeutic monoclonal antibodies (mAbs) against EGFR, which is expressed in almost 50% of GBM and GBM CSC. Our results indicate that CD16^158V^, but not CD16^158F^ CR engineered T cells incubated with cetuximab (anti‐EGFRmAb), induced the elimination of GBM‐derived CSC through a caspase‐3 dependent mechanism, and produced high amounts of TNFα and IFNγ upon the recognition of target cells. These findings provided strong in vitro evidence for the potential use of Fcγ‐CRs T cells for cancer immunotherapy (data published Online, www.annals‐oncology‐research.com).

### 4.5 CAR‐T cells and bispecific antibodies

The CAR‐T cells and bispecific antibodies (bsAbs) share a common mechanism of action where they act specifically to redirect T cells to specific TAA via the use of antibody fragments. Bispecific T cell engager (BiTE) is a subclass of bsAb that is composed of two different ScFvs derived from two separate mAbs. The first ScFv is designed to identify/bind to a specific TAA, while the second one recognizes the CD3ζ on T cells ^49^, ^50^ (Figure 1). The two variables ScFvs are interconnected by a small fusion linker that facilitates the interaction between tumor cells and T cells in an immunologic synapse.^51^ BiTE‐mediated interaction between tumor cells and T cells induce the secretion of cytokines IFNγ, TNF‐a, IL‐2, IL‐4, IL‐6, and IL‐10,^52^ and lysis of the target tumor cells occurs through perforin‐mediated delivery of granzyme B leading to calcium‐dependent proteolytic activation of intracellular caspases which results in tumor cell death.^53^, ^54^ The first clinical trial for testing the efficacy of BiTE was implemented in 2001 and has shown that CD19/CD3 targeted BiTEs (Blinatumomab, MT103) were safe and efficacious in treating ALL and B non‐Hodgkin's lymphoma (NHL) in phase I/II trials.^55^, ^56^ The efficacy of the BiTE format has been evaluated against multiple TAAs that include CD19, CD20, EpCAM, EGFR, MUC‐1, CEA, and HER2. For the solid tumor, EpCAM‐targeting MT110 has been tested in a phase I clinical trial for solid tumors including gastric, colorectal, ovarian, breast, prostate, and small cell lung cancer.^44^ For GBM, Choi et al^57^ have developed CAR‐T cells expressing both the GBM‐associated EGFRvIII, and a BiTE against EGFR. The developed CART. BiTE cells eliminated heterogeneous tumors in mouse models of glioblastoma.^57^

CAR‐T cells therapy that has been directed to a single GBM‐associated antigen was associated with antigen scape in the preclinical models of glioblastoma. In order to overcome this challenge, several studies have used CAR‐T to target surface GBM‐associated antigens, such as HER2, IL13Rα2, EphA2, and EGFRvIII. Simultaneous targeting of more than one GBM‐associated antigen has been suggested to enhance the functionality of CAR‐T cells. Bivalent CAR and tandem CAR were developed to overexpress either two distinct or single bivalent/trivalent CAR molecules, respectively, with the aim to increase the targeting capacity of CAR‐T cells.^28^, ^31^ In GBM patients, Bielamowicz et al^31^ have assessed the feasibility of killing nearly 100% of tumor cells treated in a cohort of GBM patients by determining the frequency of three targetable GBM‐antigens. Co‐targeting HER2, IL13Rα2, and EphA2 have been found to overcome interpatient variability by a tendency to capture nearly 100% of tumor cells in most tumors tested in this cohort. Given the heterogeneous expression of antigens on glioblastomas, Hegde et al^28^ created a CAR‐That joins a HER2‐binding ScFv and an IL13Rα2‐binding IL‐13 mutein to make a tandem CAR exodomain (TanCAR) and a CD28.ζ endodomain. In a murine glioblastoma model, TanCAR‐T cells mitigated antigen escape, displayed enhanced antitumor efficacy, and improved animal survival.

### 4.6 CARS and microenvironment simultaneous targeting, checkpoints

Expression of immune checkpoint inhibitors (ICPIs) such as PD‐1 in the GBM tumor microenvironment is one of the major challenges against the translation of CAR‐T from bench to clinic. In patients with glioblastoma treated with CAR‐T cells targeting EGFRvIII, in situ evaluation of the tumor environment demonstrated increased expression of inhibitory molecules and infiltration by regulatory T cells.^34^ CAR‐T and PD‐1 checkpoint blockade exhibit higher killing efficiency in vitro and showed more effective and persistent therapeutic effects on glioblastoma and led to a significantly increased number of tumor‐infiltrating lymphocytes (TILs) in the mouse model.^58^

### 4.7 Safety of CAR‐T therapy for GBM

In a recent pilot, phase I trial was conducted using a third‐generation CAR. Eighteen patients were treated with final infusion products ranging from 6.3 × 10 to 2.6 ×10 anti‐EGFRvIII CAR‐T cells. One treatment‐related mortality has been recorded after the administration of highest dose level, and all patients developed expected transient hematologic toxicities from preparative chemotherapy. Administration of anti‐EGFRvIII CAR‐transduced T cells did not demonstrate a clinically meaningful effect in patients with glioblastoma multiforme in this phase I pilot trial.^42^

Two main common toxicities have been previously recorded for CAR‐T directed to CD19 which are cytokine release syndrome and neurologic toxicity.^59^, ^60^ Manifestation of systematic CRS in GBM patients is not expected or observed as GBM patients are not bearing large tumor burdens as in the case of blood malignancies. However, given the potential for the occurrence of catastrophic localized inflammation, and the enclosed intracranial space, careful attention should be given to the possibility of localized cytokine release in any patient who developed new neurologic symptoms in the first month after CAR‐T‐EGFRvIII cell infusion. To overcome the potential of developing neurological toxicity symptoms, siltuximab along with corticosteroids was described. Siltuximab binds soluble IL‐6 and therefore was hypothesized to be safer for reducing CNS exposure and the ability to cross blood–brain barrier.^34^ Multiple or split dosing regimens have been used previously to reduce the risk of infusion‐related toxicities.^61^, ^62^ In patients treated with EGFRvIII targeting CAR‐T cells, five patients had a decrease of EGFRvIII expression and one subject was found to have a heterogeneous expression of EGFRvIII between different regions of the tumor. A similar down‐regulation of the target has also been reported in one patient who underwent surgery after treatment with IL13Ra2‐targeting CAR‐T cells for glioblastoma.^33^

Another safety concerning issue is the potential of CAR‐T cells to direct GBM‐associated antigens know to be expressed in normal tissue. Successful targeting of GBM‐associated antigen depends on the selection of antigens that are overexpressed in GBM cells, and minimally expressed in normal tissues. This would guard against the induction of toxicities that might happen due to a reaction with normal vital cells/organs. For instance, the possibility of cross‐reactivity with wild‐type EGFR was tested extensively in silico, in vitro, and in skin‐grafted xenogeneic models. No evidence of cross‐reactivity to wild‐type EGFR was recorded in this clinical setting.^63^

### 4.8 Mechanism of action of CAR‐T cell therapy for GBM

The mechanism by which the apparent tumor responses or growth delay in CAR‐T cell‐treated GBM are multifactorial. This cannot be attributed to the therapy but could instead result from differences in the natural history of disease between patients. Although there are no direct ways to demonstrate the actual killing of tumor cells by CART in situ, previous clinical^34^ and preclinical data ^63^ suggest that CAR‐T‐EGFRvIII cells induce their action by antigen‐directed cytolysis after crossing the blood–brain barrier. In another study, a marked decrease in the EGFRvIII^+^ cells was observed following the standard‐of‐care chemoradiation therapy,^64^ and thus the observed decrease of EGFRvIII + cells was not entirely attributable to the CART therapy. In addition, it has been found that the expression of EGFRvIII appears to fluctuate over time, which might indicate the existence of a stem cell pool of GBM cells. CAR‐T EGFRvIII therapy may be able to modify a subpopulation of GBM stem cells,^65^ and EGFRvIII appears to be focally expressed with both spatial and temporal variations illustrating the heterogeneity of EGFRvIII expression and diverse mechanisms of regulation.^66^

Besides its potential directed cytolysis and modulation of GBM stem cell pools, CAR‐T‐EGFRvIII cells may also target the tumor microenvironment. Following CAR‐T‐ EGFRvIII infusion, trafficking of CAR‐T cells to the brain tumor was observed within the first 2 weeks of infusion. In addition, a much greater influx of non‐transduced, polyclonal T cells was also found within the brain tissue suggesting a secondary response by non‐CAR‐expressing T cells.^34^

Another potential mechanism by which CAR‐T‐EGFRvIII may work is through the induction of epitope spreading which results in the recognition of native or mutated tumor antigens. In this regard, the presence of lymphocytes within malignant gliomas can be a positive prognostic indicator of survival.^67^, ^68^ In addition, CAR‐T‐EGFRvIII infusion was associated with the up‐regulation of the expression of other immunosuppressive molecules such as IDO1, PD‐L1, and IL‐10. This also suggests the possibility of synergy between CAR‐T cells and inhibition of IDO1 with small‐molecule drugs and/or the PD1/PD‐L1 axis with checkpoint blocking antibodies.^34^

### 4.9 Brain lymphatics and trafficking of CAR‐T through the blood–brain barrier

Trafficking of CAR‐T lymphocytes through the blood–brain barrier might also be challenging although responses have been seen in hematologic malignancies with central nervous system (CNS) involvement.^69^ There is sufficient evidence of immune checkpoint therapy for melanoma brain metastases, for example, indicating that the BBB is no hindrance to the entry of therapeutic T cells. A combined PD‐1/CTLA‐4 blockade dramatically (∼14‐fold) increased the trafficking of CD8^+^ T cells to the brain mainly through the upregulation of T cell entry receptors intercellular adhesion molecule 1 and vascular adhesion molecule 1 on tumor vasculature. This study elucidated that the inhibition of PD‐1/CTLA‐4 could augment the recruitment, activation, and release of extracranial CD8^+^ T cells, suggesting the potential application of this protocol to enhance the immune therapy‐enhancing strategy in metastatic brain cancer.^70^

The long lasting believes for the lack of classical lymphatic drainage system in the central nervous system (CNS) has recently been challenged by the study of Louveau et al^71^ who discovered functional lymphatic vessels lining the dural sinuses. These structures express that all of the molecular hallmarks of lymphatic endothelial cells are able to carry both fluid and immune cells from the cerebrospinal fluid and are connected to the deep cervical lymph nodes. The discovery of the CNS lymphatic system, and previous demonstration of potential trafficking and recruitment of CD8^+^ T cells to the brain tissue following from PD‐1/CTLA‐4 inhibition may shed new light on the mechanism by which CAR‐T could target GBM tumor cells within the brain microenvironment.^71^

## 5 CURRENTLY ACTIVE CAR‐T CLINICAL TRIALS

The safety and efficacy of CAR‐T cell therapy for GBM are currently under investigation in clinical trials using a variety of CAR‐T constructs. These trials highlight the important role of CAR‐T cell technology that may play in the future treatment of brain cancers (Table S1). In China, 20 GBM patients were enrolled to assess the safety and efficacy of CAR‐T cells directed against the EGFRvIII. The EGFRvIII CAR‐T cells were generated using a lentiviral vector incorporating two CAR constructs, including an EGFRvIII; plus another truncated EGFR (tEGFR) CAR, used for in vivo tracking and ablation of CAR‐T cells. No outcome of this trial has yet been reported (NCT02844062). In an ongoing pilot phase I study, the safety and efficacy of NKG2D CAR‐T cell therapy are being evaluated in patients with relapsed and/or refractory glioblastoma, and the outcome is expected to be released by 2023 (NCT04717999).

Interestingly, 70% of GBM patients have been reported to express B7‐H3, which is not expressed in normal tissues. Of note, the efficacy of CAR‐T therapy for CNS tumor has been found to be variable and dependent on several factors, not only in terms of the site and route of their administrations but also with regard to their combination with other therapeutic modalities, including chemo‐and radiotherapy. In an ongoing clinical trial, the safety and tolerability of intratumoral/intracerebroventricular injection of a combination of B7‐H3 CAR‐T and temozolomide (TMZ) are being evaluated, wherein the B7‐H3 CAR‐T has been injected in between temozolomide cycles. The outcomes of this study are scheduled to be released by 2022 (NCT04385173). Combined treatment with EGFRvIII CAR‐T cells and TMZ is ongoing in another phase I clinical trial (NCT02664363). Another study is underway to test the combined treatment of CAR‐T following radiosurgery, in which anti‐EGFRvIII CAR‐T cells have been administered to patients with recurrent GBM after stereotactic radiosurgery (NCT03283631).

The efficacy of CAR‐T therapy has been shown to depend not only on the specificity of the CAR construct, but also on the best dose used. Whether these dose differences contribute to the safety, tolerability, and minimization of the side effects of CAR‐T therapy remains to be elucidated. An ongoing phase I clinical trial is devoted to elucidating the best dose of CAR‐T cells. It uses a chlorotoxin tumor‐targeting domain to treat patients with MPP2+ recurrent glioblastoma. A follow‐up study will be carried out up to 15 years (NCT04214392). A dose‐escalation clinical study is ongoing to evaluate the efficacy of anti‐CD147 CART cells in patients with recurrent malignant glioma. Assessment of the therapeutic efficacy will be based on measuring the serum cytokine level and CAR‐T cell number in the whole treatment session (NCT04045847).

Advances in our understanding of the mechanism of action of immune checkpoint inhibitors (ICPIs) such as nivolumab (anti‐PD‐1) and ipilimumab (anti‐CTL4A) have provided insights into the potential use of a combined immunotherapeutic protocol of CAR‐T and ICPIs for recurrent and resistant GBM. This has led to the initiation of a phase I trial to study the side effects and how well IL13Ralpha2‐CRT T cells work when given alone or together with nivolumab and ipilimumab (PD‐1 AND CTL4A) in GBM patients. Many immune checkpoint inhibitors have demonstrated marked anti‐tumor activities. The efficacy of a combined protocol of IL13Ralpha2‐CRT T cells and nivolumab is under discussion (NCT04003649), and researchers should be aware of the differences in the therapeutic efficacy of different single/combinatorial treatment regimens that have been uncovered in different clinical studies (Table S1).

PD‐L1 is overexpressed in 88% of glioblastoma. An ongoing pilot clinical study has been initiated to decipher the safety and efficacy of CAR‐T cells directed against the PD‐L1 antigen (NCT02937844). By modifying some of the immune‐modulatory genes, the efficacy of CAR‐T cell therapy for targeting both the tumor cells and the inhibitory tumor microenvironment could be exploited (NCT03170141).

Targeting IL13Ralpha2 using anti‐IL13Ralpha2 CAR‐T cells in patients with leptomeningeal disease from glioblastoma, ependymoma, or medulloblastoma is ongoing in a phase I clinical trial investigating the potential side effects that might be associated with such therapy, as well as the ability of IL13Ralpha2‐CAR‐T cells to destroy brain tumor cells in these patients (NCT04661384). Given the importance of EGFRvIII as one of the GBM‐associated antigens, targeting GBM‐specific antigens might be very interesting as a viable approach for intervention. This led to the initiation of another phase I pilot study to determine the safety and feasibility of CART‐EGFRvIII in the treatment of patients with EGFRvIII+glioblastoma (NCT02209376). An ongoing clinical trial is planned in which patients will receive IL13R alpha 2‐specific with truncated CD19‐expressing T lymphocytes (NCT02208362).

The cellular stress‐associated NKG2D pathway is known to play a crucial role in GBM cells that display a mesenchymal signature.^72^ The efficacy and safety of NKG2D‐based CAR are planned for testing within the context of an ongoing phase I trial (NCT04270461).

Most CAR‐T studies have been performed using a single TAA. Targeting more than one TAA could improve the efficacy of CAR‐T immunotherapy. The fact that more than 80% of GBMs are positive for HER2, and that cytomegalovirus (CMV) is known to exist in most people have led to the development of a clinical trial in which HER2‐CAR‐T cells were pre‐selected for their ability to recognize cytomegalovirus (CMV) (NCT01109095).

## 6 FUTURE PERSPECTIVE

CAR‐T‐based adaptive therapy for GBM targets several GBM‐specific antigens, which are not or minimally expressed in normal tissue. Despite the promising results of CAR‐T technology in hematological malignancies, CAR‐T cells for GBM are still in their earlier stage. Several GBM‐associated antigens have served as the targets of ongoing clinical trials (EGFRvIII, NKG2D, B7‐H3, CD147, IL13Ralpha2, and HER2). Most of the current clinical trials are still in phase I testing the safety and efficacy of mostly second‐generation CAR‐T cells constructed with CD28 and 4‐1BB costimulatory intracellular domains. Despite promising results, several challenges still exist before the full approval of CAR‐T cell therapy for GBM. First is the highly hostile tumor microenvironment, which presents several limitations against the implementation of CAR‐T cells. GBM is known to be formed of a genetically variable cell population of tumor cells and stem cells. The variable genetic nature of GBM makes targeting a single GBM‐associated antigen inefficient. GBM’s heterogeneity makes antigen escape a major challenge and a principal cause of failure of CAR‐T immunotherapy.^73^ Dual‐targeted CAR‐T cells have been designed to co‐target IL‐13Rα2 and HER2.^74^ Second‐generation CAR‐T cells help to alleviate the suppressive nature of GBM microenvironment, and CAR‐T cells utilizing CD28 and 4‐1BB costimulatory signaling domains help to increase T cell survival. Third‐generation CAR‐T cells designed to release several stimulatory cytokines such as IL‐12^55^ or to constitutively express CD40,^56^ help to support T cell‐mediated immune function. Another potential complementary therapeutic modality to augment CAR‐T cells is the use of anti‐ PD‐1/PD‐L1. This strategy increases the efficacy of CAR‐T cell therapy via rejuvenation of exhausted T cells that might exist within the GBM tumor microenvironments.^75^

Cytokine release syndrome (CRS) has been recorded as one of the major toxicity issues in CAR‐T cell therapy when it is used for blood malignancies. CRS occurs due to the release of several cytokines such as IL‐6 and IFN‐γ. It produces symptoms such as encephalopathy, aphasia, delirium, and seizures, due to increased inflammatory cytokine levels and endothelial dysfunction of the BBB.^75^ One promising approach to minimize the release of cytokines in CRS is the use of anti‐IL‐6 or anti‐VEGF antibodies to minimize local inflammation.

Guarding against the risk of the on‐target‐off‐tumor effect, which can have lethal consequences (such as, HER2^76^ and MAGE^77^ ) is crucial for the successful application of CAR‐T cells for solid tumors. Moreover, the use of suicide switches and controllable CAR systems is also currently being studied in hopes of minimizing potential peripheral tissue toxicity that might be fatal.^78^

Further clinical challenges such as the optimization of the dose and route of delivery as well as the way in which the efficacy of the planned CAR‐T therapy will be assessed exist. Previous clinical studies revealed that the analysis of parameters related to local changes in the inflammatory cytokine and immune cell frequencies is more effective for testing the efficacy of CAR bioactivity.^32^

Finally, the use of combined treatment against GBM seems to be more effective than a single therapy. Such a powerful combinatorial therapeutic strategy could help to improve not only the efficacy of CAR cell, but also help to minimize potential toxic effects that might be encountered in CAR‐T cell immunotherapy for GBM.

## 7 CONCLUSION

The poor prognosis of GBM following the current standard point of care (SPC) protocol, necessitates the discovery of more effective targeted therapy modalities that precisely eliminates the highly invasive and recurrent GBM cancer cells. The promising outcomes of CAR‐T cell immunotherapy have provided hope for the use of the same strategy for CNS solid tumors including GBM. Several clinical trials are ongoing to assess the safety and efficacy of CAR‐T cells using different GBM‐associated antigens such as EGFRvIII, NKG2D, B7‐H3, CD147, IL13Ralpha2, and HER2. Despite promising results at the preclinical and phase I clinical trials level, CAR‐T cell immunotherapy for GBM patients is still in its infancy. Several challenges are still posing obstacles including the heterogeneous nature of GBM cancer cells, the hostile GBM microenvironment, antigen escape, and CRS neurotoxicity. These all necessitate a cautious approach to widespread application. Studies to overcome these challenges are ongoing, including the use of bi‐ and tri‐targeted CAR‐T cells, combined radio‐& chemo‐CAR‐T cell therapy, and the application of recently demonstrated Fcγ‐CRs T cells. The last is based on the rationale of mimicking the function of innate immune cells like NK lymphocytes to bring about the destruction of tumor cells via antibody‐dependent cellular cytotoxicity (ADCC) against specific TAA. While the Fcγ‐CRs T is still currently being tested in vitro and at the pre‐clinical level, it may become one of the leading standards for treating GBM and other solid tumors.

## CONFLICT OF INTEREST

The authors declare that there is no conflict of interest regarding the publication of this article.

## DISCLOSURES

The authors have nothing to declare concerning the present study.

## Supporting information

Table S1

## Data Availability

All data are included within the manuscript.
