# Supplementary material for: Current progress in chimeric antigen receptor T cell therapy for glioblastoma multiforme
Source: Cancer Med. 2021 Jun 19;10(15):5019–30. doi: 10.1002/cam4.4064 (PMC8335808; doi:10.1002/cam4.4064)
Supplement: Supplementary file 1 — Table S1 [file CAM4-10-5019-s001.pdf]

ClinicalTrials.gov Search Results 03/13/2021

	NCT Number	Title	Other Names	Status	Conditions	Interventions	Characteristics	Population	Sponsor/ Collaborators	Funder Type	Dates	Locations
1	NCT02844062	Pilot Study of Autologous Anti-EGFRvIII CAR T Cells in Recurrent Glioblastoma Multiforme<br><br>Study Documents:	Title Acronym:<br><br>Other Ids:<br>SBNK-2016-015-01	Unknown status	• Glioblastoma Multiforme	• Biological: anti-EGFRvIII CAR T cells<br>• Drug: cyclophosphamide<br>• Drug: Fludarabine	Study Type:<br>Interventional<br><br>Phase:<br>Phase 1<br><br>Study Design:<br>• Allocation: N/A<br>• Intervention Model: Single Group Assignment<br>• Masking: None (Open Label)<br>• Primary Purpose: Treatment<br><br>Outcome Measures:<br>• Safety of infusion of autologous anti-EGFRvIII CAR T cells with cyclophosphamide and fludarabine as lymphodepleting chemotherapy in patients with recurrent glioblastoma using the NCI CTCAE V4.0 criteria.<br>• Treatment Responses Rate<br>• Overall Survival Rate<br>• Progression-free Survival Rate<br>• Persistence of CAR T cells in patients	Enrollment:<br>20<br><br>Age:<br>18 Years to 70 Years (Adult, Older Adult)<br><br>Sex:<br>All	• Beijing Sanbo Brain Hospital<br>• Marino Biotechnology Co., Ltd.	• Other<br>• Industry	Study Start:<br>July 2016<br><br>Primary Completion:<br>July 2018<br><br>Study Completion:<br>July 2019<br><br>First Posted:<br>July 26, 2016<br><br>Results First Posted:<br>No Results Posted<br><br>Last Update Posted:<br>July 26, 2016	• Sanbo Brain Hospital Capital Medical University, Beijing, China

	NCT Number	Title	Other Names	Status	Conditions	Interventions	Characteristics	Population	Sponsor/ Collaborators	Funder Type	Dates	Locations
2	NCT04717999	Pilot Study of NKG2D CAR-T in Treating Patients With Recurrent Glioblastoma<br>Study Documents:	Title Acronym:<br>Other Ids: UBP-P02-3001-GBM	Not yet recruiting	•Recurrent Glioblastoma	•Biological: NKG2D CAR-T	Study Type: Interventional<br>Phase: Not Applicable<br>Study Design:<br>•Allocation: N/A<br>•Intervention Model: Single Group Assignment<br>•Masking: None (Open Label)<br>•Primary Purpose: Treatment<br>Outcome Measures:<br>•Number of Participants who experience a Dose-Limiting Toxicity (DLT)<br>•Overall Response Rate<br>•Progression-free survival	Enrollment: 20<br>Age: 20 Years to 70 Years (Adult, Older Adult)<br>Sex: All	•UWELL Biopharma	•Industry	Study Start: September 1, 2021<br>Primary Completion: September 30, 2023<br>Study Completion: December 31, 2023<br>First Posted: January 22, 2021<br>Results First Posted: No Results Posted<br>Last Update Posted: January 22, 2021	
3	NCT04385173	Pilot Study of B7-H3 CAR-T in Treating Patients With Recurrent and Refractory Glioblastoma<br>Study Documents:	Title Acronym:<br>Other Ids: SAHZJU-BP102	Recruiting	•Recurrent Glioblastoma<br>•Refractory Glioblastoma	•Drug: B7-H3 CAR-T<br>•Drug: Temozolomide	Study Type: Interventional<br>Phase: Phase 1<br>Study Design:<br>•Allocation: N/A<br>•Intervention Model: Single Group Assignment<br>•Masking: None (Open Label)<br>•Primary Purpose: Treatment<br>Outcome Measures:<br>•Incidence and type of adverse events<br>•Maximum tolerated dose (MTD)<br>•Overall survival (OS)<br>•Progression-free survival (PFS)<br>•The pharmacokinetics (PK) of B7-H3 CAR-T<br>•Disease response (ORR, CR, PR, DOR)	Enrollment: 12<br>Age: 18 Years to 75 Years (Adult, Older Adult)<br>Sex: All	•Second Affiliated Hospital, School of Medicine, Zhejiang University<br>•BoYuan RunSheng Pharma (Hangzhou) Co., Ltd.	•Other	Study Start: June 1, 2020<br>Primary Completion: May 1, 2022<br>Study Completion: July 1, 2022<br>First Posted: May 12, 2020<br>Results First Posted: No Results Posted<br>Last Update Posted: May 12, 2020	•the Second Affiliated Hospital of Zhejiang University School of Medicine, Hangzhou, Zhejiang, China

	NCT Number	Title	Other Names	Status	Conditions	Interventions	Characteristics	Population	Sponsor/ Collaborators	Funder Type	Dates	Locations
4	NCT03726515	CART-EGFRvIII + Pembrolizumab in GBM<br>Study Documents:	Title Acronym:<br>Other Ids: 831706, UPCC 13318	Completed	• Glioblastoma	• Biological: CART-EGFRvIII T cells<br>• Biological: Pembrolizumab	Study Type: Interventional<br>Phase: Phase 1<br>Study Design:<br>• Allocation: N/A<br>• Intervention Model: Single Group Assignment<br>• Masking: None (Open Label)<br>• Primary Purpose: Treatment<br>Outcome Measures:<br>• Number of subjects with treatment-related adverse events, using NCI CTCAE v5.0.<br>• Overall survival Rate<br>• Progression-free survival (PFS)<br>• Objective response rate (ORR)	Enrollment: 7<br>Age: 18 Years and older (Adult, Older Adult)<br>Sex: All	• University of Pennsylvania	• Other	Study Start: March 11, 2019<br>Primary Completion: February 27, 2021<br>Study Completion: February 27, 2021<br>First Posted: October 31, 2018<br>Results First Posted: No Results Posted<br>Last Update Posted: March 3, 2021	• Abramson Cancer Center of the University of Pennsylvania, Philadelphia, Pennsylvania, United States

	NCT Number	Title	Other Names	Status	Conditions	Interventions	Characteristics	Population	Sponsor/ Collaborators	Funder Type	Dates	Locations
5	NCT04077866	B7-H3 CAR-T for Recurrent or Refractory Glioblastoma<br><br>Study Documents:	Title Acronym:<br><br>Other Ids: SAHZJU-RCT-BP102	Recruiting	• Recurrent Glioblastoma<br>• Refractory Glioblastoma	• Drug: Temozolomide<br>• Biological: B7-H3 CAR-T	Study Type: Interventional<br><br>Phase:<br>• Phase 1<br>• Phase 2<br><br>Study Design:<br>• Allocation: Randomized<br>• Intervention Model: Parallel Assignment<br>• Masking: None (Open Label)<br>• Primary Purpose: Treatment<br><br>Outcome Measures:<br>• Overall survival (OS)<br>• Incidence and type of adverse events<br>• Maximum tolerated dose (MTD)<br>• Progression-free survival (PFS)<br>• Peak Concentration (Cmax) of B7-H3 CAR-T<br>• Area under the concentration versus time curve (AUC) of B7-H3 CAR-T<br>• Disease response (ORR, CR, PR, DOR)	Enrollment: 40<br><br>Age: 18 Years to 75 Years (Adult, Older Adult)<br><br>Sex: All	• Second Affiliated Hospital, School of Medicine, Zhejiang University<br>• Ningbo Yinzhou People's Hospital<br>• Huizhou Municipal Central Hospital<br>• BoYuan RunSheng Pharma (Hangzhou) Co., Ltd.	• Other	Study Start: May 1, 2022<br><br>Primary Completion: June 1, 2024<br><br>Study Completion: July 1, 2024<br><br>First Posted: September 4, 2019<br><br>Results First Posted: No Results Posted<br><br>Last Update Posted: May 19, 2020	• the Second Affiliated Hospital of Zhejiang University School of Medicine, Hangzhou, Zhejiang, China<br>• Huzhou Central Hospital, Huzhou, Zhejiang, China<br>• Ningbo Yinzhou People's Hospital, Ningbo, Zhejiang, China

	NCT Number	Title	Other Names	Status	Conditions	Interventions	Characteristics	Population	Sponsor/ Collaborators	Funder Type	Dates	Locations
6	NCT04214392	Chimeric Antigen Receptor (CAR) T Cells With a Chlorotoxin Tumor-Targeting Domain for the Treatment of MPP2+ Recurrent or Progressive Glioblastoma<br><br>Study Documents:	Title Acronym:<br><br>Other Ids:<br>•19309<br>•NCI-2019-08393	Recruiting	•Recurrent Glioblastoma<br>•Recurrent Malignant Glioma<br>•Recurrent WHO Grade II Glioma<br>•Recurrent WHO Grade III Glioma	•Biological: Chlorotoxin (EQ)-CD28-CD3zeta-CD19t-expressing CAR T-lymphocytes	Study Type:<br>Interventional<br><br>Phase:<br>Phase 1<br><br>Study Design:<br>•Allocation: N/A<br>•Intervention Model: Single Group Assignment<br>•Masking: None (Open Label)<br>•Primary Purpose: Treatment<br><br>Outcome Measures:<br>•Dose limiting toxicity (DLT)<br>•Chimeric antigen receptor (CAR) T cell<br>•Endogenous T cell<br>•Cytokine levels in TCF, PB and CSF<br>•Progression free survival time<br>•Disease response<br>•Overall survival (OS)<br>•CAR T cells detected in tumor tissue<br>•Chlorotoxin-targeted antigen expression levels in tumor tissue<br>•Biomathematical modeling of tumor growth	Enrollment:<br>36<br><br>Age:<br>18 Years and older (Adult, Older Adult)<br><br>Sex:<br>All	•City of Hope Medical Center<br>•National Cancer Institute (NCI)	•Other<br>•NIH	Study Start:<br>February 26, 2020<br><br>Primary Completion:<br>February 6, 2023<br><br>Study Completion:<br>February 6, 2023<br><br>First Posted:<br>January 2, 2020<br><br>Results First Posted:<br>No Results Posted<br><br>Last Update Posted:<br>May 1, 2020	•City of Hope Medical Center, Duarte, California, United States

	NCT Number	Title	Other Names	Status	Conditions	Interventions	Characteristics	Population	Sponsor/ Collaborators	Funder Type	Dates	Locations
7	NCT02664363	EGFRvIII CAR T Cells for Newly-Diagnosed WHO Grade IV Malignant Glioma<br><br>Study Documents:<br>• Study Protocol and Statistical Analysis Plan	Title Acronym: ExCeL<br><br>Other Ids: Pro00069444	Terminated	• Glioblastoma<br>• Gliosarcoma	• Biological: EGFRvIII CAR T cells	Study Type: Interventional<br><br>Phase: Phase 1<br><br>Study Design:<br>• Allocation: N/A<br>• Intervention Model: Single Group Assignment<br>• Masking: None (Open Label)<br>• Primary Purpose: Treatment<br><br>Outcome Measures:<br>• Maximally Tolerated Dose<br>• Number of Patients Who Experienced a Dose-limiting Toxicity (DLT)	Enrollment: 3<br><br>Age: 18 Years to 80 Years (Adult, Older Adult)<br><br>Sex: All	• Gary Archer Ph.D.<br>• Duke University	• Other	Study Start: February 1, 2017<br><br>Primary Completion: September 25, 2018<br><br>Study Completion: September 12, 2019<br><br>First Posted: January 27, 2016<br><br>Results First Posted: July 24, 2020<br><br>Last Update Posted: July 24, 2020	• The Preston Robert Tisch Brain Tumor Center at Duke, Durham, North Carolina, United States
8	NCT04045847	CD147-CART Cells in Patients With Recurrent Malignant Glioma.<br><br>Study Documents:	Title Acronym:<br><br>Other Ids: Chen Zhinan-2	Recruiting	• Recurrent Glioblastoma<br>• CD147 Positive	• Biological: CD147-CART	Study Type: Interventional<br><br>Phase: Early Phase 1<br><br>Study Design:<br>• Allocation: N/A<br>• Intervention Model: Single Group Assignment<br>• Masking: None (Open Label)<br>• Primary Purpose: Treatment<br><br>Outcome Measures:<br>• Incidence and type of adverse events induced by CD147-CART<br>• DLT and MTD of CD147-CART cell<br>• Clinical Activity of CD147-CART cell<br>• CD147-CART detection in Peripheral Blood	Enrollment: 31<br><br>Age: 18 Years to 65 Years (Adult, Older Adult)<br><br>Sex: All	• Xijing Hospital	• Other	Study Start: May 30, 2019<br><br>Primary Completion: October 30, 2020<br><br>Study Completion: May 30, 2022<br><br>First Posted: August 6, 2019<br><br>Results First Posted: No Results Posted<br><br>Last Update Posted: May 7, 2020	• National Translational Science Center for Molecular Medicine & Department of Cell Biology, Xi'an, Shaanxi, China

	NCT Number	Title	Other Names	Status	Conditions	Interventions	Characteristics	Population	Sponsor/ Collaborators	Funder Type	Dates	Locations
9	NCT04003649	IL13Ralpha2-Targeted Chimeric Antigen Receptor (CAR) T Cells With or Without Nivolumab and Ipilimumab in Treating Patients With Recurrent or Refractory Glioblastoma<br><br>Study Documents:	Title Acronym:<br><br>Other Ids:<br>• 18251<br>• NCI-2018-02764<br>• R01CA236500	Recruiting	• Recurrent Glioblastoma<br>• Refractory Glioblastoma	• Biological: IL13Ralpha2-specific Hinge-optimized 4-1BB-co-stimulatory CAR/Truncated CD19-expressing Autologous TN/ MEM Cells<br>• Biological: Ipilimumab<br>• Biological: Nivolumab<br>• Other: Quality-of-Life Assessment<br>• Other: Questionnaire Administration	Study Type:<br>Interventional<br><br>Phase:<br>Phase 1<br><br>Study Design:<br>• Allocation: Randomized<br>• Intervention Model: Parallel Assignment<br>• Masking: None (Open Label)<br>• Primary Purpose: Treatment<br><br>Outcome Measures:<br>• Incidence of adverse events<br>• Dose-limiting toxicity (DLT)<br>• Feasibility (neoadjuvant therapy)<br>• Feasibility (adjuvant therapy)<br>• Survival<br>• T cell levels<br>• Cytokine levels in TCF, PB, and CSF<br>• Disease response<br>• Time to progression<br>• Overall survival (OS)<br>• and 7 more	Enrollment:<br>60<br><br>Age:<br>18 Years to 75 Years (Adult, Older Adult)<br><br>Sex:<br>All	• City of Hope Medical Center<br>• National Cancer Institute (NCI)	• Other<br>• NIH	Study Start:<br>September 26, 2019<br><br>Primary Completion:<br>December 1, 2022<br><br>Study Completion:<br>December 1, 2022<br><br>First Posted:<br>July 1, 2019<br><br>Results First Posted:<br>No Results Posted<br><br>Last Update Posted:<br>December 9, 2020	• City of Hope Medical Center, Duarte, California, United States

	NCT Number	Title	Other Names	Status	Conditions	Interventions	Characteristics	Population	Sponsor/ Collaborators	Funder Type	Dates	Locations
10	NCT04661384	Brain Tumor-Specific Immune Cells (IL13Ralpha2-CAR T Cells) for the Treatment of Leptomeningeal Glioblastoma, Ependymoma, or Medulloblastoma<br><br>Study Documents:	Title Acronym:<br><br>Other Ids:<br>• 19497<br>• NCI-2020-06010<br>• P30CA033572	Recruiting	• Leptomeningeal Metastases	• Biological: IL13Ralpha2-specific Hinge-optimized 41BB-co-stimulatory CAR Truncated CD19-expressing Autologous T-Lymphocytes	Study Type:<br>Interventional<br><br>Phase:<br>Phase 1<br><br>Study Design:<br>• Allocation: N/A<br>• Intervention Model: Single Group Assignment<br>• Masking: None (Open Label)<br>• Primary Purpose: Treatment<br><br>Outcome Measures:<br>• Incidence of adverse events<br>• Overall survival<br>• CAR T cell levels detected in tumor cyst fluid (TCF), peripheral blood (PB), and cerebrospinal fluid (CSF)<br>• Endogenous T cell levels detected in tumor cyst fluid (TCF), peripheral blood (PB), and cerebrospinal fluid (CSF)<br>• Cell phenotype detected in tumor cyst fluid (TCF), peripheral blood (PB), and cerebrospinal fluid (CSF)<br>• Cytokine levels in PB, TCF and CSF<br>• Disease response<br>• Time to progression<br>• CAR T and endogenous cells detected in tumor tissue<br>• IL13Raphs2 antigen expression levels in tumor tissue.<br>• Biomathematical Modeling of tumor growth<br>• Biomathematical Modeling of perfusion/diffusion	Enrollment:<br>30<br><br>Age:<br>18 Years and older (Adult, Older Adult)<br><br>Sex:<br>All	• City of Hope Medical Center<br>• National Cancer Institute (NCI)	• Other<br>• NIH	Study Start:<br>February 15, 2021<br><br>Primary Completion:<br>December 15, 2022<br><br>Study Completion:<br>December 15, 2022<br><br>First Posted:<br>December 10, 2020<br><br>Results First Posted:<br>No Results Posted<br><br>Last Update Posted:<br>January 27, 2021	• City of Hope Medical Center, Duarte, California, United States

	NCT Number	Title	Other Names	Status	Conditions	Interventions	Characteristics	Population	Sponsor/ Collaborators	Funder Type	Dates	Locations
11	NCT02209376	Autologous T Cells Redirected to EGFRVIII-With a Chimeric Antigen Receptor in Patients With EGFRVIII+ Glioblastoma<br><br>Study Documents:	Title Acronym:<br><br>Other Ids: UPCC 35313, 820381	Terminated	• Patients With Residual or Reccurent EGFRvIII+ Glioma	• Biological: CART-EGFRvIII T cells	Study Type: Interventional<br><br>Phase: Phase 1<br><br>Study Design:<br>• Allocation: N/A<br>• Intervention Model: Single Group Assignment<br>• Masking: None (Open Label)<br>• Primary Purpose: Treatment<br><br>Outcome Measures: Number of adverse events	Enrollment: 11<br><br>Age: 18 Years and older (Adult, Older Adult)<br><br>Sex: All	• University of Pennsylvania<br>• University of California, San Francisco	• Other	Study Start: November 18, 2014<br><br>Primary Completion: April 4, 2018<br><br>Study Completion: April 4, 2018<br><br>First Posted: August 5, 2014<br><br>Results First Posted: No Results Posted<br><br>Last Update Posted: March 5, 2019	• UCSF, San Francisco, California, United States<br>• Abramson Cancer Center of the University of Pennsylvania, Philadelphia, Pennsylvania, United States
12	NCT02937844	Pilot Study of Autologous Chimeric Switch Receptor Modified T Cells in Recurrent Glioblastoma Multiforme<br><br>Study Documents:	Title Acronym:<br><br>Other Ids: SBNK-2016-016-01	Unknown status	• Glioblastoma Multiforme	• Biological: Anti-PD-L1 CSR T cells<br>• Drug: Cyclophosphamide<br>• Drug: Fludarabine	Study Type: Interventional<br><br>Phase: Phase 1<br><br>Study Design:<br>• Allocation: N/A<br>• Intervention Model: Single Group Assignment<br>• Masking: None (Open Label)<br>• Primary Purpose: Treatment<br><br>Outcome Measures:<br>• Number of Adverse Events related to CSR T cell infusion<br>• Treatment Responses Rate<br>• Overall Survival Rate<br>• Progression-free Survival Rate	Enrollment: 20<br><br>Age: 18 Years to 70 Years (Adult, Older Adult)<br><br>Sex: All	• Beijing Sanbo Brain Hospital<br>• Marino Biotechnology Co., Ltd.	• Other<br>• Industry	Study Start: July 2016<br><br>Primary Completion: July 2018<br><br>Study Completion: July 2019<br><br>First Posted: October 19, 2016<br><br>Results First Posted: No Results Posted<br><br>Last Update Posted: October 19, 2016	• Sanbo Brain Hospital Capital Medical University, Beijing, China

	NCT Number	Title	Other Names	Status	Conditions	Interventions	Characteristics	Population	Sponsor/ Collaborators	Funder Type	Dates	Locations
13	NCT03283631	Intracerebral EGFR-vIII CAR-T Cells for Recurrent GBM<br><br>Study Documents:	Title Acronym: INTERCEPT<br><br>Other Ids:<br>• Pro00083828<br>• 5P50CA190991-03	Suspended	• Recurrent Glioblastoma<br>• Recurrent Gliosarcoma	• Biological: EGFRvIII-CARs	Study Type: Interventional<br><br>Phase: Phase 1<br><br>Study Design:<br>• Allocation: N/A<br>• Intervention Model: Single Group Assignment<br>• Masking: None (Open Label)<br>• Primary Purpose: Treatment<br><br>Outcome Measures:<br>• Determination of Maximum Tolerated Dose (MTD)<br>• Assessment of T Cell trafficking within the brain tumor<br>• Assessment of T cell trafficking systemically<br>• Median survival<br>• Median progression-free survival	Enrollment: 24<br><br>Age: 18 Years and older (Adult, Older Adult)<br><br>Sex: All	• Gary Archer Ph.D.<br>• National Cancer Institute (NCI)<br>• Duke Cancer Institute<br>• Duke University	• Other<br>• NIH	Study Start: May 30, 2018<br><br>Primary Completion: December 31, 2021<br><br>Study Completion: December 31, 2022<br><br>First Posted: September 14, 2017<br><br>Results First Posted: No Results Posted<br><br>Last Update Posted: April 22, 2020	• Duke University Medical Center, Durham, North Carolina, United States

	NCT Number	Title	Other Names	Status	Conditions	Interventions	Characteristics	Population	Sponsor/ Collaborators	Funder Type	Dates	Locations
14	NCT03170141	Immunogene-modified T (IgT) Cells Against Glioblastoma Multiforme<br><br>Study Documents:	Title Acronym:<br><br>Other Ids:<br>GIMI-IRB-17003	Enrolling by invitation	• Glioblastoma Multiforme of Brain<br>• Glioblastoma Multiforme	• Biological: Antigen-specific IgT cells	Study Type:<br>Interventional<br><br>Phase:<br>Phase 1<br><br>Study Design:<br>• Allocation: N/A<br>• Intervention Model: Single Group Assignment<br>• Masking: None (Open Label)<br>• Primary Purpose: Treatment<br><br>Outcome Measures:<br>• Safety of infusion of autologous IgT cells with cyclophosphamide and fludarabine as lymphodepleting chemotherapy in patients with recurrent glioblastoma using the NCI CTCAE V4.0 criteria.<br>• Treatment response rate of recurrent glioblastoma<br>• Overall survival Rate<br>• Progression-free survival rate<br>• Persistence and proliferation of IgT cells in patients<br>• Production of specific immune check point modulatory proteins	Enrollment:<br>20<br><br>Age:<br>1 Year to 80 Years (Child, Adult, Older Adult)<br><br>Sex:<br>All	• Shenzhen Geno-Immune Medical Institute	• Other	Study Start:<br>May 31, 2019<br><br>Primary Completion:<br>August 1, 2019<br><br>Study Completion:<br>December 31, 2022<br><br>First Posted:<br>May 30, 2017<br><br>Results First Posted:<br>No Results Posted<br><br>Last Update Posted:<br>March 3, 2021	• Shenzhen Geno-immune Medical Institute, Shenzhen, Guangdong, China<br>• Department of Neurosurgery, Shenzhen Hospital, Southern Medical University, Shenzhen, Guangdong, China

	NCT Number	Title	Other Names	Status	Conditions	Interventions	Characteristics	Population	Sponsor/ Collaborators	Funder Type	Dates	Locations
15	NCT04270461	[NKG2D-based CAR T-cells Immunotherapy for Patient With r/r NKG2DL+ Solid Tumors]<br><br>Study Documents:	Title Acronym:<br><br>Other Ids: JiujiangUH	Withdrawn	•Hepatocellular Carcinoma<br>•Glioblastoma<br>•Medulloblastoma<br>•Colon Cancer	•Biological: NKG2D-based CAR T-cells	Study Type: Interventional<br>Phase: Phase 1<br>Study Design:<br>•Allocation: N/A<br>•Intervention Model: Single Group Assignment<br>•Masking: None (Open Label)<br>•Primary Purpose: Treatment<br>Outcome Measures:<br>•Number of Participants with severe cytokine release syndrome(CRS) as a Measure of Safety and Tolerability.<br>•Copies numbers of CAR<br>•overall survival (OS)<br>•Progress Free Survival (PFS)<br>•Duration of response, (DoR)	Enrollment: 0<br>Age: 18 Years to 75 Years (Adult, Older Adult)<br>Sex: All	•Jiujiang University Affiliated Hospital<br>•KAEDI	•Other	Study Start: March 17, 2020<br>Primary Completion: October 17, 2020<br>Study Completion: October 17, 2020<br>First Posted: February 17, 2020<br>Results First Posted: No Results Posted<br>Last Update Posted: October 22, 2020	•Affiliated hospital of jiujiang university, Jiujiang, Jiangxi, China
16	NCT03392545	[Combination of Immunization and Radiotherapy for Malignant Gliomas (InSituVac1)]<br><br>Study Documents:	Title Acronym: InSituVac1<br><br>Other Ids: B0011	Recruiting	•High Grade Glioma<br>•Glioblastoma<br>•Glioma of Brainstem<br>•Glioma, Malignant	•Combination Product: Combined immune adjuvants and radiation	Study Type: Interventional<br>Phase: Phase 1<br>Study Design:<br>•Allocation: N/A<br>•Intervention Model: Single Group Assignment<br>•Masking: None (Open Label)<br>•Primary Purpose: Treatment<br>Outcome Measures:<br>•Incidence of Treatment-related Adverse Events<br>•Progression-free Survival<br>•Overall Survival	Enrollment: 30<br>Age: 18 Years to 65 Years (Adult, Older Adult)<br>Sex: All	•Beijing Tiantan Hospital<br>•Duke University	•Other	Study Start: April 1, 2018<br>Primary Completion: April 1, 2020<br>Study Completion: June 1, 2020<br>First Posted: January 8, 2018<br>Results First Posted: No Results Posted<br>Last Update Posted: July 11, 2019	•Beijing Tiantan Hospital, Beijing, Beijing, China

	NCT Number	Title	Other Names	Status	Conditions	Interventions	Characteristics	Population	Sponsor/ Collaborators	Funder Type	Dates	Locations
17	NCT01454596	CAR T Cell Receptor Immunotherapy Targeting EGFRvIII for Patients With Malignant Gliomas Expressing EGFRvIII<br><br>Study Documents:<br>• Study Protocol and Statistical Analysis Plan<br>• Informed Consent Form	Title Acronym:<br><br>Other Ids:<br>• 110266<br>• 11-C-0266	Completed	• Malignant Glioma<br>• Glioblastoma<br>• Brain Cancer<br>• Gliosarcoma	• Biological: Epidermal growth factor receptor(EGFRv)III Chimeric antigen receptor (CAR) transduced PBL<br>• Drug: Aldesleukin<br>• Drug: Fludarabine<br>• Drug: Cyclophosphamide	Study Type: Interventional<br><br>Phase:<br>• Phase 1<br>• Phase 2<br><br>Study Design:<br>• Allocation: Non-Randomized<br>• Intervention Model: Sequential Assignment<br>• Masking: None (Open Label)<br>• Primary Purpose: Treatment<br><br>Outcome Measures:<br>• Number of Treatment Related Adverse Events<br>• Progression Free Survival<br>• Number of Patients With an Objective Response<br>• Circulating Chimeric Antigen Receptor (CAR+) Cells in Peripheral Blood at 1 Month Post Treatment<br>• Number of Participants With Serious and Non-serious Adverse Events Assessed by the Common Terminology Criteria in Adverse Events (CTCAE v4.0)	Enrollment: 18<br><br>Age: 18 Years to 70 Years (Adult, Older Adult)<br><br>Sex: All	• National Cancer Institute (NCI)<br>• National Institutes of Health Clinical Center (CC)	• NIH	Study Start: May 16, 2012<br><br>Primary Completion: November 1, 2018<br><br>Study Completion: January 17, 2019<br><br>First Posted: October 19, 2011<br><br>Results First Posted: August 21, 2019<br><br>Last Update Posted: August 21, 2019	• National Institutes of Health Clinical Center, 9000 Rockville Pike, Bethesda, Maryland, United States

	NCT Number	Title	Other Names	Status	Conditions	Interventions	Characteristics	Population	Sponsor/ Collaborators	Funder Type	Dates	Locations
18	NCT01109095	CMV-specific Cytotoxic T Lymphocytes Expressing CAR Targeting HER2 in Patients With GBM<br><br>Study Documents:	Title Acronym: HERT-GBM<br><br>Other Ids: H-24487 - HERT GBM	Completed	• Glioblastoma Multiforme (GBM)	• Biological: HER.CAR CMV-specific CTLs	Study Type: Interventional<br><br>Phase: Phase 1<br><br>Study Design:<br>• Allocation: N/A<br>• Intervention Model: Single Group Assignment<br>• Masking: None (Open Label)<br>• Primary Purpose: Treatment<br><br>Outcome Measures:<br>• Number of subjects with dose limiting toxicity after CTL infusion<br>• Decrease in tumor after the CTL infusion<br>• Area under the growth curves (AUC) over time for T cell frequencies.<br>• Impact of gene modified CTL on CMV-specific T lymphocyte immune response	Enrollment: 16<br><br>Age: Child, Adult, Older Adult<br><br>Sex: All	• Baylor College of Medicine<br>• The Methodist Hospital System<br>• Center for Cell and Gene Therapy, Baylor College of Medicine	• Other	Study Start: October 2010<br><br>Primary Completion: June 2014<br><br>Study Completion: March 7, 2018<br><br>First Posted: April 22, 2010<br><br>Results First Posted: No Results Posted<br><br>Last Update Posted: April 25, 2019	• Houston Methodist Hospital, Houston, Texas, United States<br>• Texas Children's Hospital, Houston, Texas, United States

	NCT Number	Title	Other Names	Status	Conditions	Interventions	Characteristics	Population	Sponsor/ Collaborators	Funder Type	Dates	Locations
19	NCT02208362	Genetically Modified T-cells in Treating Patients With Recurrent or Refractory Malignant Glioma<br>Study Documents:	Title Acronym:<br>Other Ids:<br>• 13384<br>• NCI-2014-01488<br>• P30CA033572<br>• R01FD005129	Recruiting	• Recurrent Glioblastoma<br>• Recurrent Malignant Glioma<br>• Recurrent WHO Grade II Glioma<br>• Recurrent WHO Grade III Glioma<br>• Refractory Glioblastoma<br>• Refractory Malignant Glioma<br>• Refractory WHO Grade II Glioma<br>• Refractory WHO Grade III Glioma	• Biological: IL13Ralpha2-specific Hinge-optimized 4-1BB-co-stimulatory CAR/Truncated CD19-expressing Autologous TN/MEM Cells<br>• Biological: IL13Ralpha2-specific Hinge-optimized 41BB-co-stimulatory CAR Truncated CD19-expressing Autologous T-Lymphocytes<br>• Other: Laboratory Biomarker Analysis<br>• Procedure: Magnetic Resonance Imaging<br>• Procedure: Magnetic Resonance Spectroscopic Imaging<br>• Other: Quality-of-Life Assessment	Study Type: Interventional<br>Phase: Phase 1<br>Study Design:<br>• Allocation: Non-Randomized<br>• Intervention Model: Parallel Assignment<br>• Masking: None (Open Label)<br>• Primary Purpose: Treatment<br>Outcome Measures:<br>• Incidence of grade 3 toxicity<br>• Incidence of dose limiting toxicity (DLT), graded using National Cancer Institute (NCI) Common Toxicity Criteria for Adverse Events (CTCAE) version 4.0<br>• Incidence of toxicities, graded using National Cancer Institute (NCI) Common Toxicity Criteria for Adverse Events (CTCAE) version 4.0 as well as the modified neurological grading system<br>• Changes in largest length of tumor<br>• Changes in cytokine levels<br>• Changes in chimeric antigen receptor (CAR) T levels<br>• Progression free survival (PFS)<br>• Disease response by the Response Assessment in Neuro-Oncology criteria<br>• Disease response (stratum 1 and 2)<br>• CAR T cell detection (stratum 1 and 2)<br>• and 6 more	Enrollment: 92<br>Age: 12 Years to 75 Years (Child, Adult, Older Adult)<br>Sex: All	• City of Hope Medical Center<br>• National Cancer Institute (NCI)<br>• Food and Drug Administration (FDA)	• Other<br>• NIH<br>• U.S. Fed	Study Start: May 18, 2015<br>Primary Completion: January 18, 2022<br>Study Completion: January 18, 2022<br>First Posted: August 5, 2014<br>Results First Posted: No Results Posted<br>Last Update Posted: February 11, 2021	• City of Hope Comprehensive Cancer Center, Duarte, California, United States
